# Persistent shunt dependency and very late shunt failure in a 3-year-old boy with idiopathic intracranial hypertension (IIH)

**DOI:** 10.1007/s00381-016-3316-7

**Published:** 2016-12-17

**Authors:** Anne Henriette Paulsen, Bernt Johan Due-Tønnessen, Tryggve Lundar, Karl-Fredrik Lindegaard

**Affiliations:** 1 Department of Neurosurgery, Oslo University Hospital, Postboks 4950 Nydalen, 0424 Oslo, Norway; 2 University of Oslo, Oslo, Norway

Dear Editor:

This letter reports an unexpected clinical experience of persistent shunt dependency in a 3-year-old boy who underwent cerebrospinal fluid (CSF) diversion for IIH.

In 1937, Walter Dandy published a series of 22 patients treated surgically for intracranial hypertension without a tumor [1]. Among his cases treated with subtemporal decompression during a period of 10 years, there were three children. Later on, this condition with papilledema, headache, and visual disturbances was called benign intracranial hypertension, and many of them could be managed favorably by medical treatment [2]. In more severe cases, it soon became clear that this obscure disease is not always that benign [3]. In severe cases, the term malignant pseudotumor was introduced [4].

Later on, the term idiopathic intracranial hypertension (IIH) was introduced [5].

Neurosurgical treatment with CSF diversion has been performed in severe cases where the response to medical treatment has been unsatisfactory, or as primary treatment in patients with severe visual affection [4, 5]. Unlike the situation for hydrocephalic children treated with CSF shunts who for the most become shunt dependent, clinical results on long-term shunt dependency in IIH are unavailable.

We have treated a boy with IIH giving us a remarkable clinical experience:

In 1989, a 3-year-old boy was admitted with a short history of lost vision for 36 hours, reduced pupil reactivity, ataxia, and poor general condition. Fundoscopy demonstrated choked discs, and cerebral MRI was normal including unobstructed venous outflow. Lumbar puncture revealed normal CSF composition and increased CSF pressure, but the intracranial pressure (ICP) level was difficult to measure due to lack of cooperation. A lumbar infusion test during general anesthesia demonstrated increased CSF opening pressure as well as slightly increased outflow resistance.

Due to the dramatic clinical symptoms with complete visual loss, corticosteroid treatment was implemented, and an acute shunt procedure was performed during the same general anesthesia. A proximal catheter was introduced into cisterna magna and connected to a low pressure Holter valve with diversion to the peritoneal cavity.

His vision gradually reappeared within days, and after 1 week, there was normal vision and pupillary reactivity to light. Some ataxia and clumsy motor function persisted for weeks, but after 6 months his clinical condition was quite normal. During the first 2 years of treatment, he experienced a few episodes with headache, ataxia, and diplopia (sixth nerve paresis) which resolved spontaneously within a couple of days or after pumping on the Holter valve. At the age of 5 years (1991), he demonstrated episodes of overdrainage in the upright position, which subsided after implementation of an ASD (anti-syphon device) distal to the valve.

**Fig. 1:**
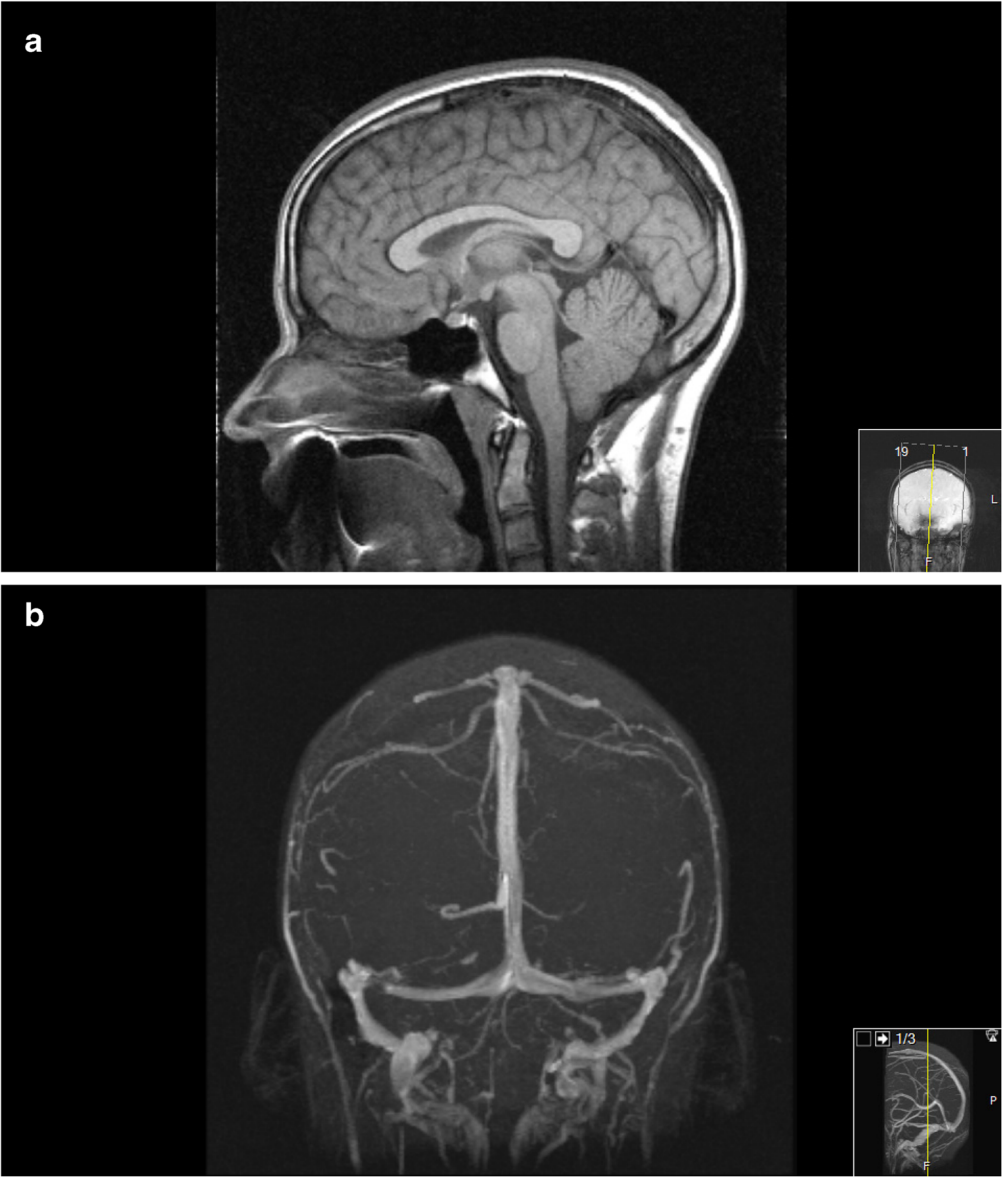
Normal MRI including venous outflow taken in 2006 during episode of shunt failure

He grew up in a family with a large repertoire of sport activities. At the age of 19 years, he joined the technical university of Norway and fulfilled a master’s degree. During his university studies (in 2006), he became acutely ill with signs of increased ICP (headache and vomiting). There was no choked discs, but lumbar puncture revealed markedly increase CSF pressure level (50 cm H_2_O) and no signs of infection. Once more, MRI was normal (Fig. 1). After a shunt revision (LP-shunt), his clinical condition normalized within a few days. In 2009, he experienced shunt failure once more, again followed by rapidly improvement after shunt revision. Thereafter, he has been working full time and has been clinically quite well for another 7 years.

The present case with a very rewarding clinical result indicates that this 30 year-old man in excellent condition most likely is permanently a shunt dependent.

Neurosurgical treatment by CSF diversion is well established in IIH patients with severe visual affection as well as in cases with unsatisfactory response to medical treatment. The present case report describes the full return of vision after complete blindness in a 3 year-old boy. While the initial response to shunt implantation is usually good, shunt failure as well as symptoms of overdrainage has been reported.

When the clinical result is good over years, as in this case, it is difficult to know if the shunt is still functioning or the underlying condition has normalized spontaneously.

Shunt implantation in children with severe IIH raises the question of making these children permanently shunt dependent [3]. While some authors have addressed this question, no cases with late or very late shunt failure has been reported.

This case with 27 years follow-up, indicate that individuals shunted for IIH can be persistently dependent on their shunt, and may experience acute shunt failure even after many years of treatment.
